# A Fatal Case of Primary Amoebic Meningoencephalitis (PAM) Complicated with Diabetes Insipidus (DI): A Case Report and Review of the Literature

**DOI:** 10.1155/2020/4925819

**Published:** 2020-07-24

**Authors:** Muhammad Zain Mushtaq, Saad Bin Zafar Mahmood, Adil Aziz

**Affiliations:** Department of Medicine, Aga Khan University Hospital, Stadium Road, Karachi, Pakistan

## Abstract

*Naegleria fowleri* is a highly infective free-living amoeba usually isolated from soil and fresh water and is primarily found to infect the central nervous system (CNS) resulting in primary amoebic meningoencephalitis (PAM). PAM as a cause of meningitis is often overlooked for other, more common causes of meningitis. Despite all the advances in antimicrobial therapy and supportive care systems, the mortality rate of this rare infection remains above 95% with the bulk of the cases being found in developed countries. We are presenting a case of a 44-year-old male with fever, worsening headache, and generalized weakness. Lumbar puncture showed a raised leucocyte count of 1100/*µ*L with predominant polymorphonuclear cells, and wet mount prep for *Naegleria fowleri* was positive further confirmed with PCR. The patient was started Intravenous (IV) and intrathecal amphotericin-B, Per Oral (PO) miltefosine, IV rifampin, IV fluconazole, and IV dexamethasone. However, the patient started producing urine at 300–500 ml/hour. The patient's sodium levels increased from 144 to 175 mmol/L in 12 hours with raised serum osmolality and decreased urine osmolality and urine sodium. The patient was started on PO desmopressin of 0.2 micrograms twice daily after which his urine output dropped to 60–80 ml/hour and sodium decreased from 175 to 162 and, later 155 mmol/L; however, the patient expired. PAM is a rare and extremely fatal illness, but with increasing incidence now being reported in developing countries as a result of better diagnostics. DI is a very rare complication reported in these patients leading to poor outcome. The complication of diabetes insipidus (DI) has not been extensively studied in patients having PAM. Only three cases have been reported with this complication. No mechanism has been mentioned in the literature behind the development of DI in these patients, and no study has mentioned laboratory details of DI as mentioned in this report.

## 1. Introduction

*Naegleria fowleri* is a highly infective free-living amoeba usually isolated from soil and fresh water mostly during summer times when higher temperatures provide a conductive environment for its growth and proliferation [1, 2]. *Naegleria fowleri* was first found in 1899 and is primarily found to infect the central nervous system (CNS) resulting in primary amoebic meningoencephalitis (PAM) [3]. Infections with *Naegleria fowleri* are primarily associated with exposure to fresh water during recreational and religious (ablution) activities; however, inadequately chlorinated water supply at homes and in swimming pools are now fast becoming possible causes for infections with the organism [1, 3]. The organism enters the body via the nasal cavity when contaminated water is deeply inhaled. It follows the olfactory nerve, moving along the cribriform plate and invades the CNS via the olfactory neuroepithelium and cribriform plate, causing an infection clinically resembling acute bacterial meningitis (ABM) [1, 2]. PAM, as a cause of meningitis, is often overlooked for other, more common causes of meningitis [4]. Despite all the advances in antimicrobial therapy and supportive care systems, the mortality rate of this rare infection remains above 95% with the bulk of the cases being found in developed countries as infections in developing countries remain mostly undiagnosed [3]. We describe here a case of *Naegleria fowleri* complicated with diabetes insipidus (DI). To the best of our knowledge, only three such cases have been reported in the literature worldwide but all of them being either children or adolescent [5–7].

## 2. Case Presentation

A previously healthy 44-year-old male with no prior comorbidities presented to emergency with a 2-day history of fever, worsening headache, and generalized weakness. He had no history of exposure to pool water. Examination revealed a Glasgow coma scale (GCS) of 14/15 with no focal deficit or neck stiffness. Laboratory workup was sent which showed leukocytosis 13.3 × 109/L. Rest of the investigations are summarized in Table 1. Computed tomography (CT) scan brain was performed which was reported normal. Lumbar puncture was performed which showed a raised opening pressure of 20 cm of water and had a raised leucocyte count of 1100/*µ*L with predominant polymorphonuclear cells of 60% with a very high protein of 241 mg/dl and low glucose, i.e., 30 mg/dl (Table 2). The BioFire filmarray Polymerase chain reaction (PCR) was negative for bacteria and viruses, but wet mount prep for *Naegleria fowleri* was positive which was further confirmed with PCR.

The patient was started on Primary amebic meningoencephalitis (PAM) protocol which included Intravenous (IV) and intrathecal amphotericin-B, Per Oral (PO) miltefosine, IV rifampin, IV fluconazole, and IV dexamethasone. He was electively intubated and shifted to the intensive care unit (ICU). A repeat CT scan after 24 hours of hospital stay was performed which did not show any infarct or bleed but did show marked cerebral edema. He was started on mannitol 30 grams every 8 hours, which was stopped after 48 hours.

During the ICU stay, the patient remained on mechanical ventilation and was deeply sedated with agents, IV propofol and midazolam. Antiepileptic drug IV levetiracetam was added empirically, and an Electroencephalogram (EEG) was obtained which showed theta and delta slowing down with no epileptiform activity. He was kept well hydrated during the ICU stay, and continuous feeding was carried out through a nasogastric tube.

On the 3rd hospital day, the patient started producing urine at 300–500 ml/hour (he made a urine output of more than 7 liters in 24 hours). His sodium levels increased from 144 mmol/L to 175 mmol/L in 12 hours. His urea was 18 mg/dl, ionized calcium was 4.88 mg/dl, and blood sugar levels were 171 mg/dl. He was well hydrated with IV ringer lactate. Serum and urinary osmolality were performed which were 332 mosm/kg and 204 mosm/kg, respectively. Urine sodium was less than 10 mEq/L. He was started on PO desmopressin of 0.2 micrograms twice daily after which his urine output dropped to 60–80 ml/hour and sodium decreased from 175 to 162 and, later, 155 mmol/L. On the 4th hospital day, he went in asystole and died. His family had decided for Do Not Resuscitate (DNR) during the ICU admission keeping in view of the poor outcome of primary amoebic meningoencephalitis.

## 3. Discussion

The first ever case of primary amoebic meningoencephalitis (PAM) to be reported in the literature was back in 1965 by Fowler and Carter in Australia [8]. Since then, around 200 total cases have been reported worldwide with the majority of the cases being reported to have come from the United States of America (USA) approximating around 138 back in 2015 [9, 10]. The first reported case in Pakistan was in 2008 [11]. Previous studies have demonstrated that PAM has a partiality to the male gender; however, no scientific explanation is there to support it [12]. The primetime for infection with the amoeba is during summer when temperatures are high and water recreational activities are up to the maximum [1, 2]. However, as per the literature, the majority of the cases from Pakistan have exposure to *Naegleria fowleri* via tap water and not through pool water [2]. In our case report, the patient presented in April 2019; however, he had no previous history of exposure to pool water.

The presenting clinical symptoms of PAM are quite similar to those of acute bacterial meningitis such as headache, fever, nausea, and vomiting, followed by altered consciousness and seizures [1, 9]. This is also one of the reasons that majority of the cases remain undiagnosed resulting in such a low reported incidence of the disease. Our patient also presented with fever and headache; however, he did not have any neurological focal deficits. The diagnosis of *Naegleria fowleri* is predominantly based on the presence of trophozoites in the cerebrospinal fluid (CSF) detected via the trichome or giemsa stain. The other findings of CSF analysis including raised opening pressure are not very specific to PAM [9].

The complication of diabetes insipidus (DI) has not been extensively studied in patients having PAM. Only three cases have been reported with this complication [5–7]. The first reported instance was from 1969 of a 24-year-old boy who had a swimming pool exposure 6 days prior to developing symptoms and expired after 5 days of hospital stay developing DI as a complication during hospital stay. He also had a history of head trauma from an auto accident as a child but was otherwise in relatively good health [5]. The second case was from 1995 of an 11-year-old boy who presented with symptoms of fever, abdominal pain, headache, nausea, and vomiting found to have a CSF picture consistent with that of ABM and was kept on ceftriaxone [7]. However, his condition quickly deteriorated, and he also developed DI during his short of stay of 34 hours in the hospital. Later on, his kidneys and liver were donated, and no complications were noted. The latest case to observe DI in a patient suffering from PAM was from Madagascar in 2005, where a 7-year-old boy was found to develop symptoms 10–12 days after exposure and died a week later having developed myocarditis and DI as a complication during the hospital stay [6]. No mechanism has been mentioned in the literature behind the development of DI in these patients, and no study has mentioned laboratory details of DI as mentioned in this report.

The mortality of PAM is exceptionally high with only 7 survivors being reported in a study from 2015 [13]. Reports from Pakistan have also reported such high mortality rate with one study reporting two survivors and the other only reporting one [1, 2]. The treatment protocol designed by Centers for Disease Control and Prevention (CDC) includes high doses of intravenous and intrathecal amphotericin-B along with other therapies that can be useful such as rifampin, azithromycin, miltefosine, and miconazole [9, 13]. Similarly, the treatment initiated in our patient was in accordance with the guidelines from CDC.

In conclusion, PAM is a rare and extremely fatal illness, but with increasing incidence now being reported in developing countries as a result of better diagnostics. DI is a very rare complication reported in these patients leading to poor outcome. More research is needed with regards to the mechanism behind DI and to ascertain the best treatment approach in these patients.

## Figures and Tables

**Table 1:** Investigation details on day 1.

Total leucocyte count	13.6 × 109 (L)
Neutrophils	92.3%
Lymphocytes	4.3%
Monocytes	3.3%
Eosinophils	0%
Random blood sugar	147 mg/dL
Malaria parasite (ICT)	Negative
Blood culture	Negative

**Table 2:** Cerebrospinal fluid detailed report on day 1.

White blood cell count	1166 (*µ*L)
Red blood cell count	0 (*µ*L)
Gram stain	Negative
India ink	Negative
Crypotococcal antigen	Negative
Wet prep for *Naegleria*	Positive
*Naegleria fowleri* by PCR	Positive

## Data Availability

Data sharing is not applicable to this study as no datasets were generated or analyzed during the current study.
